# Idiopathic non-cirrhotic portal hypertension in a patient with *Talaromyces marneffei* infection: a case report

**DOI:** 10.1186/s12879-023-08090-6

**Published:** 2023-03-01

**Authors:** Xiuling Ye, Xin Quan, Xu Guo, Zhidong Wang, Hao Wu

**Affiliations:** Department of Gastroenterology, West China Hospital, Sichuan University, No. 37, Guo Xue Xiang, Chengdu, 610041 People’s Republic of China

**Keywords:** Idiopathic non-cirrhotic portal hypertension, *Talaromyces marneffei*, Intestinal infections, Hyper-IgE syndrome, Case report

## Abstract

**Background:**

The etiopathogenesis of idiopathic non-cirrhotic portal hypertension (INCPH) is so far poorly understood. Altered immunity, blood diseases, infections, congenital defects and drug exposure have been documented in a part of patients with INCPH owing to increased recognition of the disorder in patients with HIV, or various haematological disorders or autoimmune diseases. We aim to discuss the possible etiopathogenesis of INCPH.

**Case presentation:**

We reported that a patient with intestinal infection of *T. Marneffei* and hyper-IgE syndrome, a group of rare primary immunodeficiency disorders, was finally diagnosed with INCPH for gastroesophageal variceal bleeding. The diagnosis was mainly based on histopathological features. Transjugular intrahepatic portosystemic shunt was performed and there was no recurrence of melena during the six-month follow-up.

**Conclusion:**

In the context of immunodeficiency, INCPH may associated with intestinal infections. Thus, screening for enterogenic infection and immunological disorders in patients with unexplained portal hypertension is necessary.

## Background

Idiopathic non-cirrhotic portal hypertension (INCPH) is a rare vascular liver disease characterized by presinusoidal portal hypertension (PHT) without cirrhosis [1]. It has been diagnosed in up to 84% of patients with common variable immune deficiency, hyper-IgM syndrome, and et al., although mechanisms linking immunological abnormalities to INCPH remain unknown [2]. Abdominal infections have also been implicated as one of the triggers for the development of INCPH through repeated obstruction of small to medium portal branches. However the evidence underlying this association is mainly from epidemiological studies [2]. The etiopathogenesis is so far poorly understood. Herein, we reported that a patient with INCPH was previously diagnosed with intestinal infection of *T. Marneffei* and hyper-IgE syndrome (HIES), a group of rare primary immunodeficiency disorders [3], which had never reported in INCPH before.

## Case presentation

**Fig. 1:**
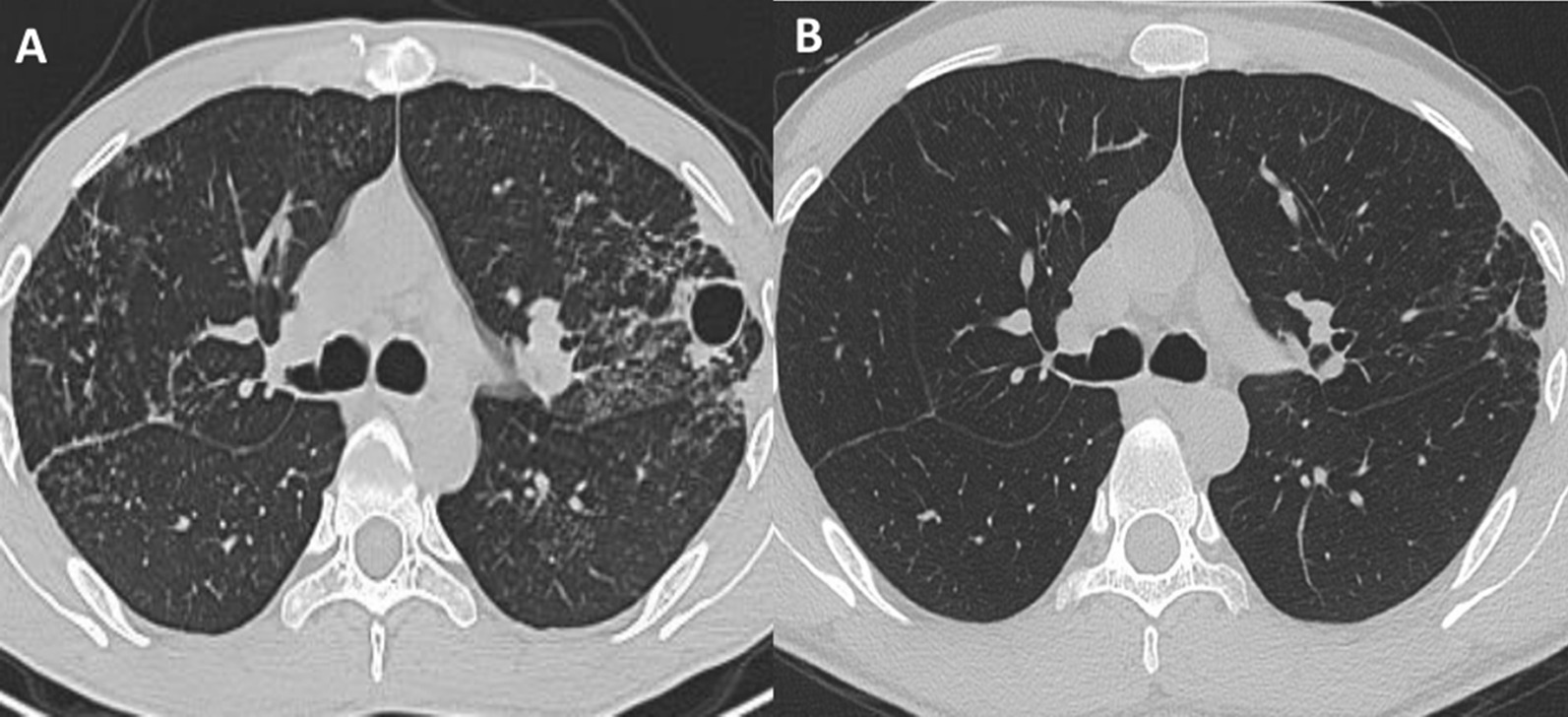
(**A**) Chest CT showed multiple infectious lesions in the upper lobe of the double lungs during the first hospitalization, which were absorbed obviously in the second hospitalization (**B**)

**Fig. 2:**
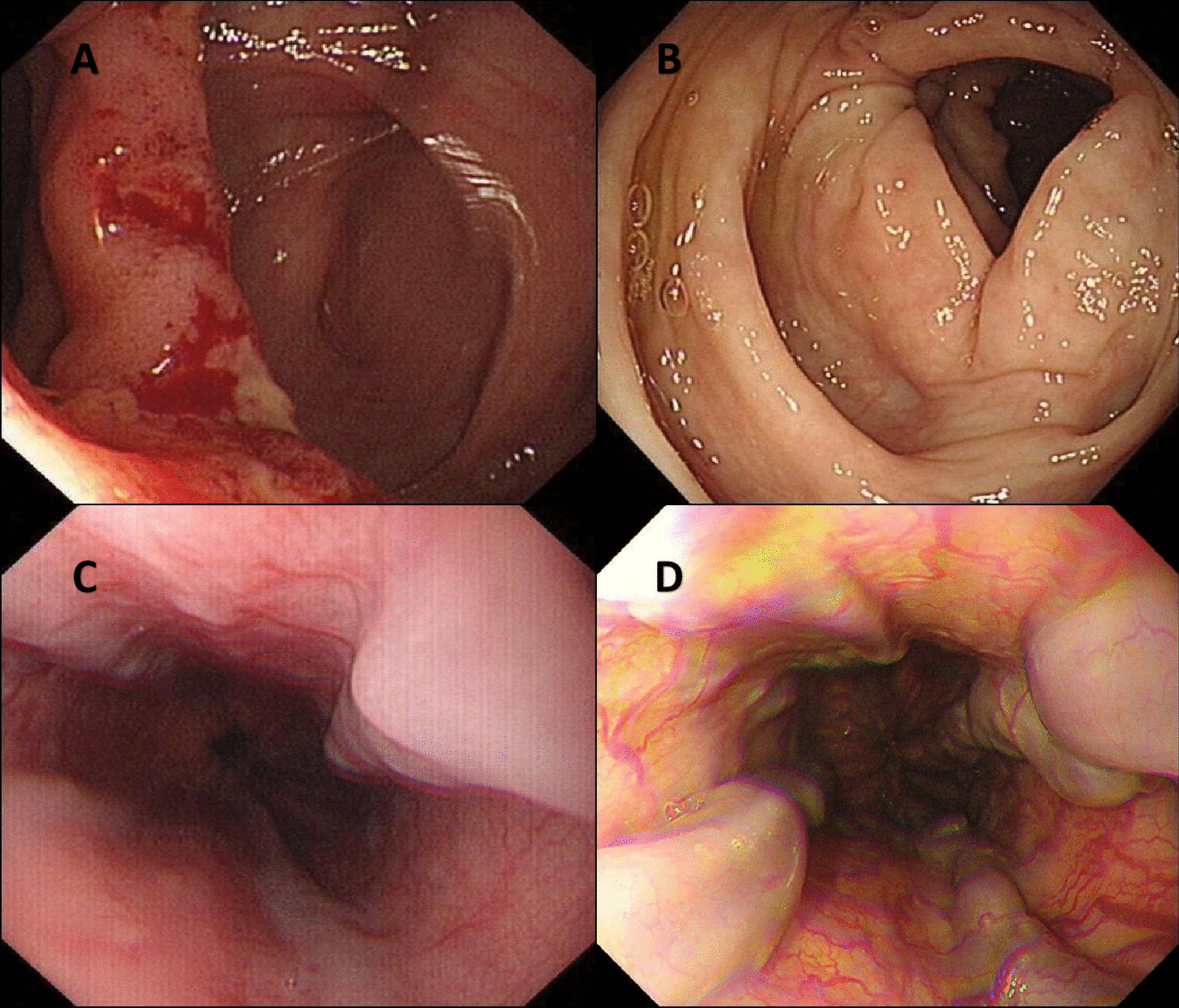
The first colonoscopy showed an irregularly shaped ulcer at the ileocecal valve (**A**), which was found to be cured after the 15-month antifungal therapy (**B**). The esophageal varices was mild (**C**) when he had first esophagogastroduodenoscopy and was proved to aggravate to the severe gastroesophageal varices with the presence of red sign 29 months later (**D**)

**Fig. 3:**
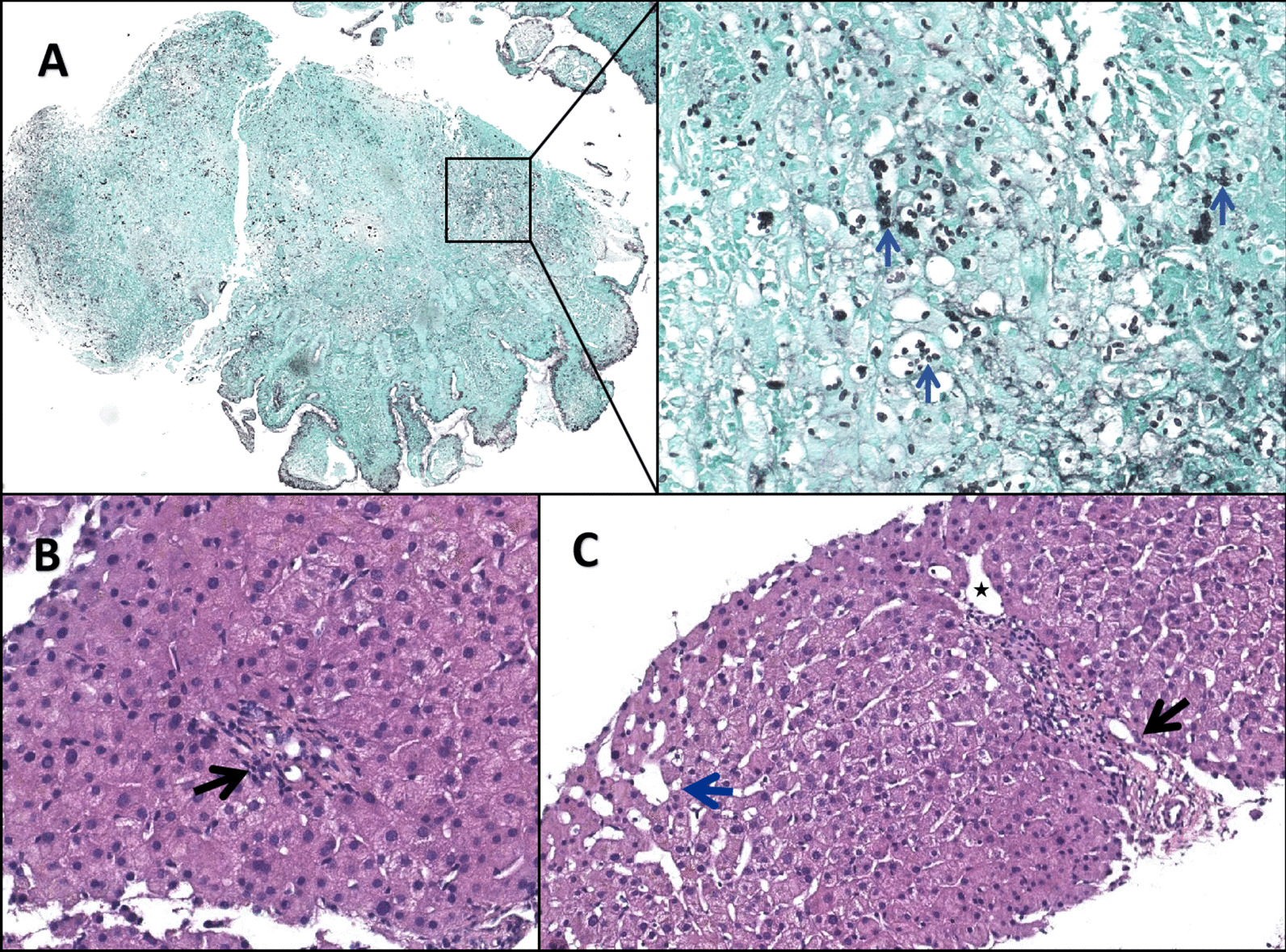
(**A**) Histopathologic examination for the ileocecal ulcer showed granulomatous inflammation with much round fungal structures (blue arrows; hexamine silver). (**B**) A fibrotic portal tract (black arrow) with obliteration of portal vein branches (H&E, magnifcation × 400). (**C**) Multiple thin walled vascular spaces in the portal tract (black arrow) with a periportal abnormal vessel (star). Compensatory enlargement of hepatic sinusoid (blue arrow) can be detected (H&E, magnifcation × 400)

A 26-year-old man was hospitalized due to a 6-month history of fever, cough, expectoration and melena. In the primary hospital, labs reported decreased hemoglobin and increased serum IgE (Table 1). Anti-HIV antibody was not detected but immunodeficiency was found in decreased lymphocyte subsets counts, especially the natural killer T (NKT). Chest computed tomography (CT) showed a pneumatocele in the left lung and bilateral infectious lesions (Fig. 1A). The endoscopy found an ulcer at the ileocecal valve (Fig. 2A) and mild esophageal varices (Fig. 2C). The results of a culture of the sputum yielded *T. Marneffei*. Periodic acid-Schiff and Hexamine silver staining of the ileocecal biopsy tissue revealed *T. Marneffei* infection (Fig. 3A). Conclusively, intestinal infection of *T. Marneffei* and hyper-IgE syndrome (HIES) were included in the diagnosis, with the score = 28 according to NIH scoring system [4] (Table 2). The whole-exon gene sequencing identified compound heterozygous mutation in *dedicator of cytokinesis-8* (*DOCK8*), the causing of HIES. He received intravenous amphoterincin B therapy (from small dose to 0.75 mg / kg/d) for 25 days and was discharged on an oral regimen of voriconazole (400 mg / d) for 15 months until the symptoms disappeared. Recheck of colonoscopy showed the ileocecal lesion was cured (Fig. 2B).

**Table 1:** Notable laboratory tests in the first and second hospitalization

Laboratory tests	First hospitalization	Second hospitalization	Reference values
Routine analysis of blood	Hemoglobin, 70 g/L	Hemoglobin, 74 g/L	130-175 g/L
	White blood cells, 4.75 × 10^9^/L	White blood cells, 2.32 × 10^9^/L	3.5-9.5 × 10^9^/L
	Neutrophils, 2.42 × 10^9^/L	Neutrophils, 1.19 × 10^9^/L	1.8-6.3 × 10^9^/L
	Platelets, 171 × 10^9^/L	Platelets, 111 × 10^9^/L	100-300 × 10^9^/L
	Hematocrit, 0.25 L/L	Hematocrit, 0.28 L/L	0.40-0.50 L/L
Blood biochemical item	Total bilirubin, 12 µmol/L	Total bilirubin, 22.7 µmol/L	5.0-28.0 µmol/L
	Alanine aminotransferase, 18 IU/L	Alanine aminotransferase, 20 IU/L	＜50 IU/L
	Aspartate aminotransferase, 36 IU/L	Aspartate aminotransferase, 25 IU/L	＜40 IU/L
	Albumin, 26.8 g/L	Albumin, 43.6 g/L	40.0-55.0 g/L
	Globulin, 46.7 g/L	Globulin, 23.9 g/L	20.0-40.0 g/L
	Alkaline phosphatase, 632 IU/L	Alkaline phosphatase, 84 IU/L	51-160 IU/L
	Γ-glutamyl transpeptidase, 321 IU/L	Γ-glutamyl transpeptidase, 47 IU/L	＜60 IU/L
	Creatinine, 84 µmol/L	Creatinine, 72 µmol/L	68-108 µmol/L
	Potassium, 4.1 mmol/L	Potassium, 3.6 mmol/L	3.5-5.3 mmol/L
	Sodium, 138.4 mmol/L	Sodium, 140.1 mmol/L	137.0-147.0 mmol/L
	Blood sugar, 4.28 mmol/L	Blood sugar, 4.59 mmol/L	3.90-5.90 mmol/L
Blood clotting	Prothrombin time, 13.5 s	Prothrombin time, 13.7 s	9.6-12.8 s
	International normalized ratio, 1.21	International normalized ratio, 1.28	0.88-1.15
	Activated partial thromboplastin time, 45.1 s	Activated partial thromboplastin time, 32.8 s	24.8-33.8 s
Erythrocyte sedimentation rate (ESR) and C-reactive protein (CRP)	ESR, 66 mm/h	ESR, 15.0 mm/h	＜21 mm/h
	CRP, 75.3 mg/L	CRP, 1.51 mg/L	＜5 mg/L
Hepatitis virus	HBsAg, 0.467 COI	HBsAg, 0.482 COI	0-0.9 COI
	HCV antibody, 0.031 COI	HCV antibody, 0.035 COI	0-0.9 COI
Immunoglobulin	IgE, 4600 IU/ml	IgE, 910 IU/ml	5.0-150.0 IU/ml
	IgG, 16.3 g/L	IgG, 13.7 g/L	8.0-15.5 g/L
	IgA, 3220 mg/L	IgA, 942 mg/L	836-2900 mg/L
	IgM, 518 mg/L	IgM, 1630 mg/L	700-2200 mg/L
NK/B/T cell count (percent)	NK cell count (percent), 14 cell/µl (1.1%)	NK cell count (percent), 84 cell/µl (8.2%)	154-768 cell/µl (9.26-23.92%)
	B cell CD19 and CD5 count (percent), 31 cell/µl (3.9%)	B cell CD19 and CD5 count (percent), 153 cell/µl (15.8%)	175-332 cell/µl (3.91-8.59%)
	CD4+ T cell count (percent), 359 cell/µl (35.6%)	CD4+ T cell count (percent), 303 cell/µl (33.7%)	471-1220 cell/µl (33.19-47.85%)

**Table 2:** NIH Scoring System of HIES with Clinical and Laboratory Tests

Clinical Findings	Points									
	0	1	2	3	4	5	6	7	8	10
Highest serum – IgE level (IU / ml)	<200	200–500			501–1000				1001–2000	>2000
Skin abscesses	None		1–2		3–4				>4	
Pneumonia (episodes over lifetime)	None		1		2		3		>3	
Parenchymal lung anomalies	Absent						Bronchiectasis		Pneumatocele	
Retained primary teeth	None	1	2		3				>3	
Scoliosis, maximum curvature	<10°		10–14°		15–20°				>20°	
Fractures with minor trauma	None				1–2				>2	
Highest eosinophil count (cell/μl)	<700			700–800			>800			
Characteristic face	Absent		Mildly present			Present				
Midline anomaly	Absent					Present				
Newborn rash	Absent				Present					
Eczema (worst stage)	Absent	Mild	Moderate		Severe					
Upper respiratory infections per year	1–2	3	4–6		>6					
Candidiasis	None	Oral	Fingernails		Systemic					
Other serious infections	None				Severe					
Fatal infection	Absent				Present					
Hyperextensibility	Absent				Present					
Lymphoma	Absent				Present					
Increased nasal width	<1 SD	1–2 SD		>2 SD						
High palate	Absent		Present							
Young – age correction	>5 yaers			2–5 years		1–2 years		≤ 1 years		

At ≥ 15 points, the subject is likely to carry an HIES genotype; at 10–14 points, the presence of an HIES genotype is indeterminate; and at <10 points, the subject is unlikely to have an HIES genotype

**Fig. 4:**
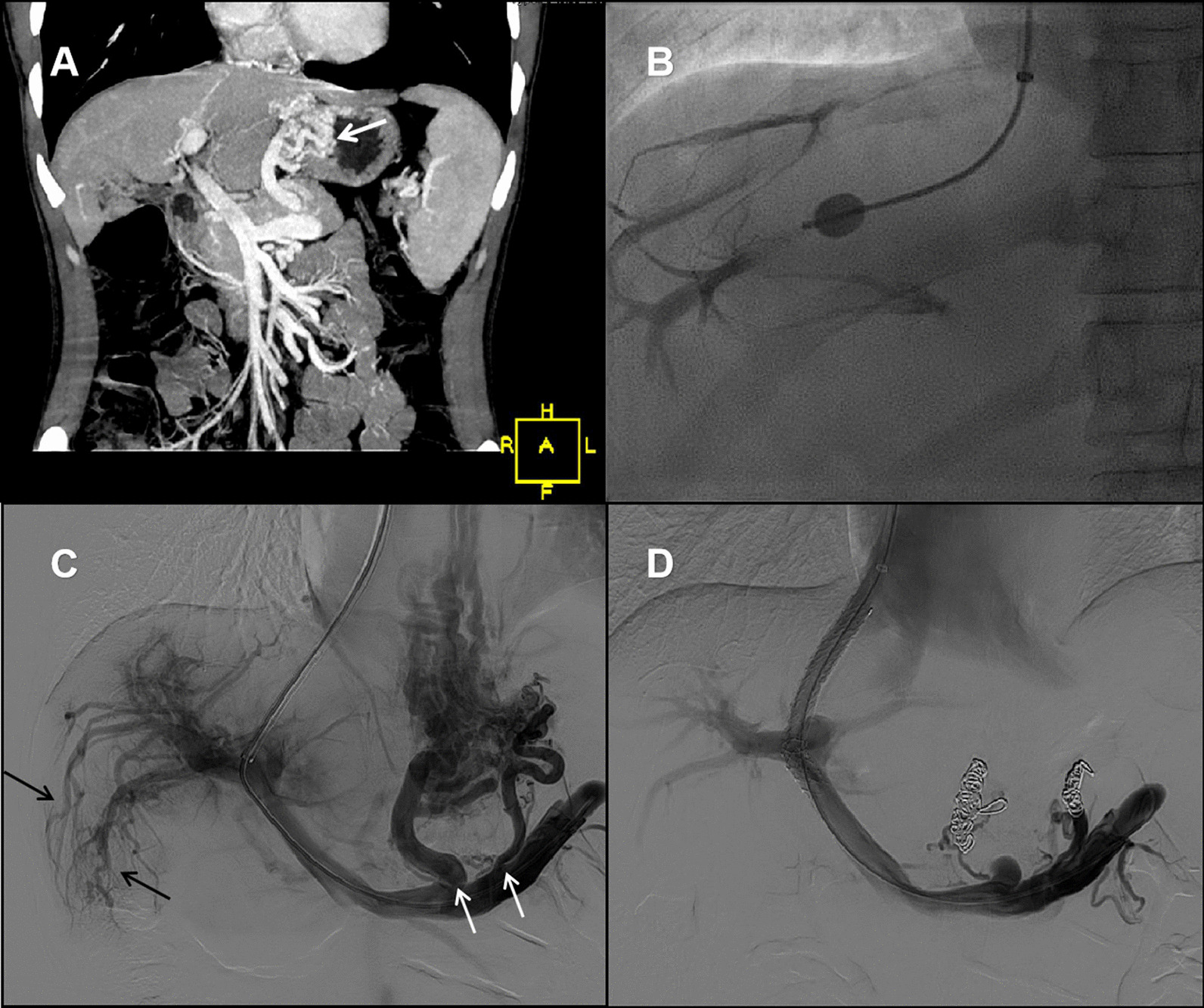
**A** Contrast-enhanced abdominal CT angiography with three-dimensional reconstruction showed a smooth liver surface, splenomegaly and gastroesophageal varices (white arrow). **B** Hepatic vein-to-vein communication was detected by hepatic venography. **C** Portography showed irregularity and tortuosity of the peripheral portal branches with much abrupt interruptions (black arrow) and avascular area beneath the liver surface. Portovenous collaterals (white arrows) derived from gastric coronary vein were also displayed. **D** Post-TIPS portography revealed embolization of collaterals and clear stent flow from portal vein to systemic circulation

However, the patient was readmitted to our hospital 14 months later because of recurrent melena. Laboratory tests showed decreased hemoglobin, decreased leukocyte, and normal liver function (Table 1). Endoscopy reveled the gastroesophageal varices significantly aggravated (Fig. 2D). Contrast-enhanced abdominal CT showed splenomegaly and gastroesophageal varices without obstruction of main portal vein or inferior vena cava (Fig. 4A). The liver biopsy was performed due to no evidence of chronic liver disease based on labs and previous history. The histopathological changes showed features of portal vein stenosis (Fig. 3B) and hyper-vascμlarized portal tracts (Fig. 3C) with out pseudolobule formation. Finally, the patient was diagnosed with INCPH.

Transjugular intrahepatic portosystemic shunt (TIPS) was performed to prevent variceal rebleeding. During the procedure, hepatic vein-to-vein communication (HVVC) was displayed via hepatic venography (Fig. 4B). Portography showed tortuosity of portal vein branches with much interruptions (Fig. 4C). The hepatic venous pressure gradient (HVPG) was detected as only 4 mmHg while the portal pressure gradient before and after stent placement were 18 mmHg and 6 mmHg, respectively. The collaterals was disappeared after TIPS (Fig. 4D) and there was no hepatic encephalopathy and recurrence of melena during the six-mounth follow-up.

## Discussion and conclusion

The diagnosis of INCPH was based on radiological examination had excluded prehepatic and posthepatic PHT (such as extrahepatic portal venous obstruction and Budd Chiari syndrome), and liver biopsy displayed portal vein branches stenosis and hyper-vascularized portal tracts, rather than severe hepatic fibrosis or pseudolobule formation [5]. HVVC and normal HVPG detected by hepatic vein catheterisation as well as the portography features also conformed with the diagnosis [6]. The etiopathogenesis of INCPH is so far poorly understood. Immunological abnormalities, including primary antibody-deficiency and HIV infection as well as autoimmune disorders seem to be associated with INCPH [1]. However, autosomal recessive HIES caused by *DOCK8* deficiency is a primary combined immunodeficiency which had never reported in INCPH before. Mutations in the *DOCK8* gene encoding a guanine nucleotide exchange factor highly expressed in lymphocytes that regulates the actin cytoskeleton were identified as the most common cause for autosomal recessive HIES [3]. *DOCK8* deficiency impairs immune cell migration, function and survival which characterized by allergic inflammation as well as susceptibility towards infections, autoimmunity and malignancy [7]. Previous studies have suggested the abnormalities of genes and specific adhesion molecules involved in lymphocyte activation in patients with INCPH [8, 9]. These suggest lymphocyte-endothelial cell cross-talk could play a role in the pathogenesis.

Other possible mechanisms of the portal vein branches damage in patients with immunological disorders includes abdominal infections, immunosuppressants, or anti-infection drugs. In our case, the anti-fungal drugs can not explain the disease because the signs of PHT appeared much earlier. It is noteworthy that the patient had an intestinal infection with *Talaromyces marneffei.* The fungus is gradually emerging in southeast Asia and southern China which HIV infection is regarded as the most underlying disease [10]. Recently *T. Marneffei* infections in non-HIV individuals are increasing whose clinical characteristics are atypical [11]. *T. Marneffei* is a primary lung pathogen that disseminates to other internal organs by lymphatic or hematogenous mechanisms [12]. In our case, there were definite evidences of the presence of *T. Marneffei* in the lung and intestine. Coupled with a close anatomical connection between the liver and the gut it can be concluded that the patient already had a disseminated disease of *T. Marneffei.* In other words, *T. Marneffei* may enter the liver through the superior mesenteric vein and cause endothelial and vascular damage. Additionally, *T. Marneffei* infection and INCPH exist in sequence, whereas the primary immunodeficiency was already present. Therefore, we hypothesized that the infection of *T. marneffei* played a major role in the pathogenesis of INCPH. As the *DOCK8* deficiency affects the long-term retention of NKT-cell in the liver [13] and may therefore increase the susceptibility to infections, the intestinal involved of *T. Marneffei* as well as previous occult intestinal infections combined with immune disorders may be the cause of INCPH in this patient [14]. The association between *T. Marneffe*i infections and INCPH has not been reported in previous studies. INCPH is more common in developing countries where bacterial infection is rampant [1, 15]. Also means that as long as it produces septic emboli which cause endothelial damage, microthrombosis, sclerosis, and obstruction of the small and medium-sized portal branches any intestinal infection leads to INCPH. This hypothesis is supported by an animal study that recurrent injection of heat killed *Escherichia coli* into portal vein can develop the rabbit model of INCPH [16].

As for treatment, the main purpose is to prevent PHT related complications for the lack of etiotropic therapy. A retrospective study including 41 patients with INCPH have demonstrated good effectiveness and safety of TIPS for PHT related complications [17]. In the current case, there is no episode of hepatic encephalopathy or abnormal liver function tests occurred after TIPS during the six-mounth follow-up. Despite the scarcity of evidence, TIPS may precede other therapies for INCPH due to preserved liver function and less post-TIPS complications.

This case demonstrated that, like other kinds of immunodeficiency, primary combined immunodeficiency can occur in INCPH. Intestinal infections may lead to further portal vein damage in such conditions. Thus, screening for enterogenic infection and immunological disorders in patients with unexplained PHT is necessary. Futher studies on mechanisms connecting intestinal infections and INCPH are needed.

## Data Availability

The data and materials that support the findings of this study are available from West China Hospital, Sichuan University, but restrictions apply to the availability of these data, which were used under license for the current study, and so are not publicly available. Data are however available from the authors upon reasonable request and with permission of West China Hospital, Sichuan University.

## Abbreviations

INCPH: Idiopathic non-cirrhotic portal hypertension
HIV: Human immunodeficiency virus
PHT: Portal hypertension
*T. Marneffei*: *Talaromyces marneffei*
HIES: Hyper-IgE syndrome
NKT: Natural killer T
CT: Computed tomography
NIH: National Institutes of Health
TIPS: Transjugular intrahepatic portosystemic shunt
HVPG: Hepatic venous pressure gradient
HVVC: Hepatic vein-to-vein communication
